# Polyethylene Glycol-Mediated Exosome Isolation: A Method for Exosomal RNA Analysis

**DOI:** 10.61186/ibj.4129

**Published:** 2024-02-24

**Authors:** Abdulwahab Teflischi Gharavi, Azadeh Niknejad, Saeed Irian, Amirabbas Rahimi, Mona Salimi

**Affiliations:** 1 Department of Cell and Molecular Sciences, Faculty of Biological Sciences, Kharazmi University, Tehran, Iran;; 2 Department of Physiology and Pharmacology, Pasteur Institute of Iran, Tehran, Iran;; 3 Molecular Medicine Department, Biotechnology Research Center, Pasteur Institute of Iran, Tehran, Iran

**Keywords:** Exosomes, Extracellular vesicles, MicroRNA

## Abstract

**Background:**

ExoRNAs offer valuable insights into their cellular origin. ExoRNA studies were faced with challenges in obtaining sufficient amounts of high-quality RNA. Herein, we aimed to compare three traditional exosome isolation methods to introduce an appropriate strategy to extract RNA from cancer-derived exosomes for further RNA analysis.

**Methods:**

Exosomes were isolated through ultracentrifugation, precipitation kit, and size exclusion column chromatography, and then characterized by DLS and TEM, followed by extracting total RNA. The quality and quantity of the extracted RNAs were assessed by a NanoDrop and 2.5% agarose gel electrophoresis.

**Results:**

Extracted exosomes displayed a similar range of size and morphology. We found that PEG-precipitation method resulted in a higher RNA yield with a 260/280 ratio of 1.9. The obtained exoRNA appeared as a smear in the agarose gel, indicative of small exoRNAs.

**Conclusion:**

We provide researchers a suitable approach to isolate exosomes based on yield and purity of exoRNA.

## INTRODUCTION

Exosomes, discrete populations of EVs ranging from 30 to 200 nm, are originated from multivesicular bodies and secreted by most cell types^[^^1^^]^. These phospholipid nanocarriers play a decisive role in cell-cell communication processes via acting as transmitters between parent cells and specific target cells. Exosomes deliver a variety of bioactive molecules, including DNA and RNAs, such as circRNAs, lncRNAs, mRNAs, to the target cells^[^^2^^]^. There is compelling evidence on exoRNAs being involved in the regulation of diverse gene expression and cellular functions of the recipient cells. These RNAs can influence normal physiological metabolic processes and also contribute to the development of the pathological abnormalities such as tumor growth, neurodegenerative conditions, and metabolic syndromes^[^^2^^,^^3^^]^. Notably, cancer cells release a large quantity of exosomes into tumor microenvironment to communicate with the targeted cells^[^^4^^]^. Hence, exoRNAs, which are known for their greater diversity compared to other exosomal molecules, provide valuable insights into the cellular origin of the exosomes. In this regard, elucidating the biological roles of the RNA molecules carried by exosomes derived from cancer cells would be beneficial for diagnostic or prognostic applications. For this purpose, exoRNA sequencing would provide a diverse array of coding and noncoding RNA molecules^[^^5^^]^.

A growing body of evidence has indicated that exosomes are rich in miRNAs^[^^6^^]^. These RNAs are short non-coding RNA molecules, typically 18-24 nucleotides in length, and play a role in regulating mRNA transcription through epigenetic mechanisms in various pathophysiological conditions^[^^7^^]^. They act by regulating mRNA stability and translation, thereby influencing the expression of numerous genes^[^^8^^]^. MiRNAs carried by cancer-derived exosomes contribute to a wide variety of cancer cell processes such as tumor proliferation, metastasis, and resistance to treatment^[^^9^^]^. In this light, some types of the miRNAs have tumor suppressing activity, while the others have a role in tumor progression. Therefore, targeting them would be a promising tool for cancer therapy depending on the type of miRNA^[^^10^^]^. Moreover, a better understanding of the roles and functions of the exo-miRNAs may facilitate the development of noninvasive, pioneering and novel therapeutic approaches for cancer^[^^11^^,^^12^^]^. 

A significant challenge in exo-miRNA studies includes insufficient yield and low purity of the extracted RNA. Various exosome isolation methods have introduced to augment both quality and quantity of the extracted RNA by improving the recovery of exosomes^[^^13^^]^. The techniques are categorized into two main groups, traditional and novel. The first group includes UC, density gradient centrifugation, immunomagentic beads, SECs, and precipitation methods using polymers such as PEG or adding salts such as sodium acetate. The second group employs novel strategies for exosome isolation, including ultrafiltration, integrated double-filtration microfluidic device, and on-chip isolation methods. Recently, a great deal of attention has been drawn towards using ultracentrifugation, SEC, and precipitation, due to the convenience, a significant recovery, and the specificity of the approach^[^^14^^]^.

In the present study, we aimed to compare three different traditional exosome isolation methods, namely ultracentrifugation, PEG precipitation, and SEC, based on the morphology and size of the exosomes, as well as the quality and quantity of the RNAs they enclose. The outcomes will help us to identify an appropriate strategy for RNA extraction from cancer-derived exosomes for further RNA analysis.

## MATERIALS AND METHODS

**Cell culture**

MCF-7 (IBRC C10082) cells were purchased form the Iranian Biological Resource Center (IBRC, Iran) and utilized as a source of cancer-derived exosomes. The cells (2.5 × 10^5^) were transferred to 75 cm^2^ cell culture flaks and cultured in DMEM (Gibco, USA) containing 10% FBS and penicillin-streptomycin (both from Gibco). After the confluency reached 80%, culture media were replaced with a DMEM containing 5% exosome-free FBS (Gibco) for 48 h. Cell viability was measured using the trypan blue exclusion dye assay.

**Exosome isolation**

**Fig. 1:**
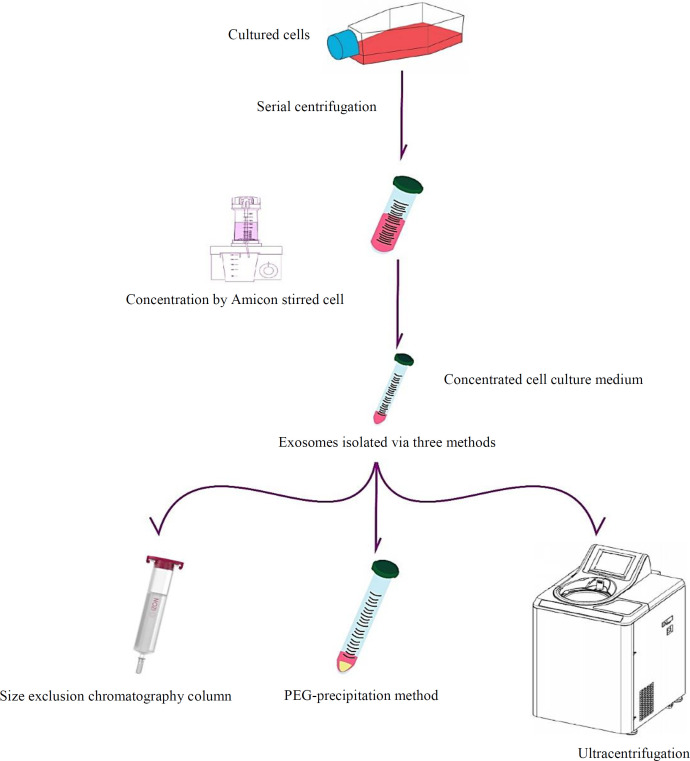
Summary of exosomes isolation. MCF-7 cells were initially harvested, and then their supernatants were collected and purified by serial centrifugations. Afterwards, the supernatant containing the exosomes were concentrated and used for exosome isolation by SEC, PEG precipitation, and UC

Following 48 h, the conditioned medium of the cultured MCF-7 cells was collected. The floating cell debris was removed after centrifugation at 1500 ×g at 4 °C for 10 min. Large EVs were excluded by centrifugation at 10,000 ×g for 30 min. The conditioned medium was then concentrated using Amicon Stirred Cell (UFSC20001, USA) and used for isolating the exosomes through three different methods: UC, PEG precipitation (EXOCIB kit, Iran) and SEC (Izon Science, New Zealand) (Fig. 1).

***Ultracentrifugation***

The concentrated medium was overlaid onto a 10.4 ml Beckman tube and centrifuged at 100,000 ×g at 4 °C for 90 min (Ti98 rotor, Beckman-Coulter, California, USA). Afterwards, the pellet was diluted in PBS and then re-centrifuged at 100,000 × g at 4 °C for 90 min. Finally, the resulted pellet was re-suspended in 200 μl of PBS and stored at 4 °C. 

***PEG precipitation***

A ratio of 1:5 of EXOCIB kit solution was added to the concentrated medium, then mixed vigorously for 5 min and incubated on a shaker at 4 °C overnight. The total mixture was vortexed for 1 min and further centrifuged at 3000 ×g at 4 °C for 40 min. The resulted pellet was resuspended in 400 μl of solution B present in the kit and stored at 4 °C for 1 day.

***Size exclusion chromatography***

The concentrated medium (500 μl) was loaded on top of a 70 nm qEV original size exclusion column. After discarding the void volume, 1.5 ml of exosome-containing fractions were recovered. The recovered exosomes were concentrated using 100 kDa cut-off filters (Amicon Ultra-50) by centrifuging at 3000 ×g at 4 °C for 30 min. The samples were aliquoted and stored at -80 °C for long storage and 4 °C until use.

**Dynamic light scattering**

Size distribution of the isolated exosomes was measured by DLS (Malvern Instruments Ltd. Nano-ZS ZEN3600, UK). For this purpose, 1 ml of the isolated exosomes filtered by a 0.22 μm syringe filter was loaded into a special cuvette. The Z-average represents the average diameter of the vesicles in nanometers. Three separate measurements were conducted for each sample using tracing analysis to measure the intensity distribution of particle sizes at a temperature of 25 °C. The Zetasizer software version 7.01 was used to collect and analyze the data. 

**Transmission electron microscopy**

TEM was applied to conduct a structural analysis of exosomes. To achieve this goal, 50 µl of the suspended exosomes was combined with 3% glutaraldehyde in a 1:1 ratio for fixation on copper grids. Subsequently, dried copper grids were examined using a Zeiss EM900 transmission electron microscope operating at 80 kV (Germany) to assess the size and morphology of the exosomes.

**Bicinconinic acid assay**

Protein concentration was determined using BCA assay (DNA Biotech, Iran). A variety of bovine serum albumin concentrations were utilized to illustrate the standard curve. A total of 25 µl of samples was loaded into each well of a 96-well plate, and 75 µl of BCA solution was then added. The plate was incubated at 60 °C for 60 min, and the absorbance was detected at 562 nm using a microplate spectrophotometer (Biotek, USA).

**RNA analyses**

***RNA extraction***

Total RNA and exoRNAs from MCF-7 cells were extracted by RNA extraction method using the TrizolEX (DNA Biotech). To this end, 1 ml of trizol reagent was added to the cells and isolated exosomes, 0.2 ml of chloroform was added to it, and then the samples were centrifuged at 12000 ×g at 4 °C for 15 min. Afterwards, the upper phase was collected, 0.5 ml of isopropanol was added and re-centrifuged at 12,000 ×g at 4 °C for 15 min. Following RNA precipitation, the RNA pellets were washed using 1 ml of 75% ethanol, centrifuged at 12,000×g at 4 °C for 2 min, and the pellets were air-dried at room temperature for 10 min. RNA pellets were dissolved in DEPC water (Sinaclon, Iran) and stored at -80 °C. 

***Quantification and qualification of RNA***

To quantify the total RNA extracted from exosomes and the MCF-7 cells, 2 µl of each sample was loaded onto a microplate, and their absorbance at 260 nm was measured by a microplate spectrophotometer (Biotek). The ratio of the absorbance at 260 nm to 280 nm was detected to assess the quality of the extracted RNA. To further explore the RNA quality, i.e. purity and integrity, a 2.5% agarose gel electrophoresis was utilized. For this aim, 5 µl of each total RNA sample was added to the agarose gel wells and electrophoresed at 100 v for 45 minutes. Subsequently, the agarose gel was documented using a Gel Doc (Uvitec, UK).

## RESULTS

**Similar size distribution and morphology using different exosomal isolation methods**

**Fig. 2:**
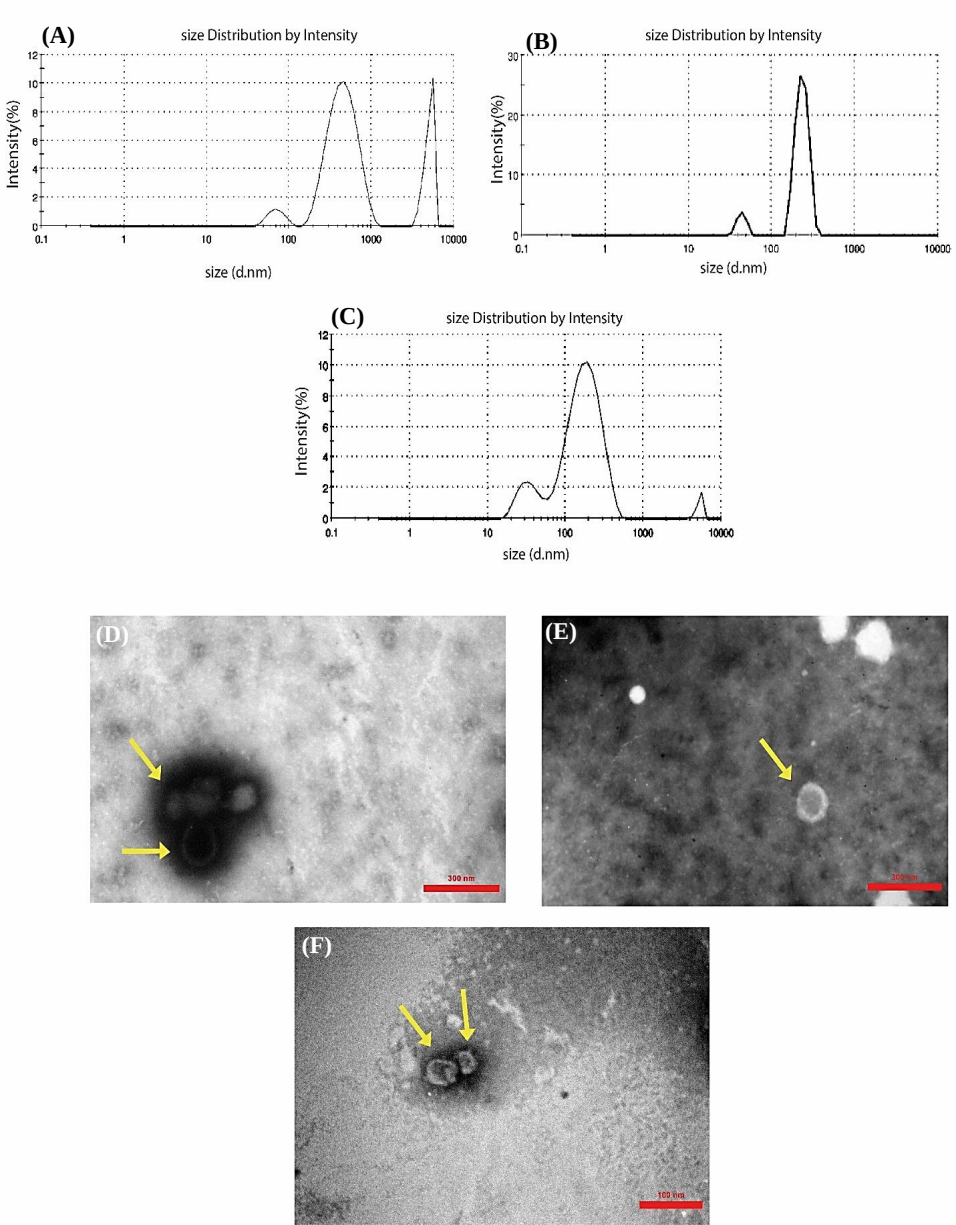
Size distribution analysis of exosomes isolated using (A) SEC, (B) UC, and (C) PEG precipitation methods by DLS in terms of intensity. TEM of negatively stained exosomes with a diameter ranged from 30 to 200 nm. Exosomes obtained from condition media via (D) UC, (E) PEG precipitation, and (F) SEC methods. Exosomes are shown by yellow arrows

DLS results showed that the average exosome size isolated by all the three methods from the cell culture medium ranged from 100 to 800 nm, whereas a small number of exosomes with an average size of more than 1000 nm was observed for the exosomes isolated by SEC and PEG precipitation techniques, which may be attributed to the concentration of the sample (Fig. 2A-2C). Consistently, the examination of exosomes using TEM analysis indicated that exosomes were intact and had a spherical shape. These findings also confirmed the same size range of exosomes as those in the DLS diagrams (Fig. 2D-2F). Exosomes were also characterized based on the presence of exosomal markers (data not shown).

**Low protein concentrations by using UC and SEC methods**

A strategy to quantify exosomes is to use their protein concentrations^[15]^. Exosomes isolated by UC and SEC methods showed protein concentrations of 34.4 ± 4.71 and 9.7 ± 2.4 µg/ml per flask, respectively, when the cells were around 80% confluent. Surprisingly, we could not measure the protein concentration of exosomes extracted by PEG precipitation method since the absorbance value was out of range of the standard curve.

**High yield and purity of exoRNA obtained by PEG precipitation**

**Fig. 3:**
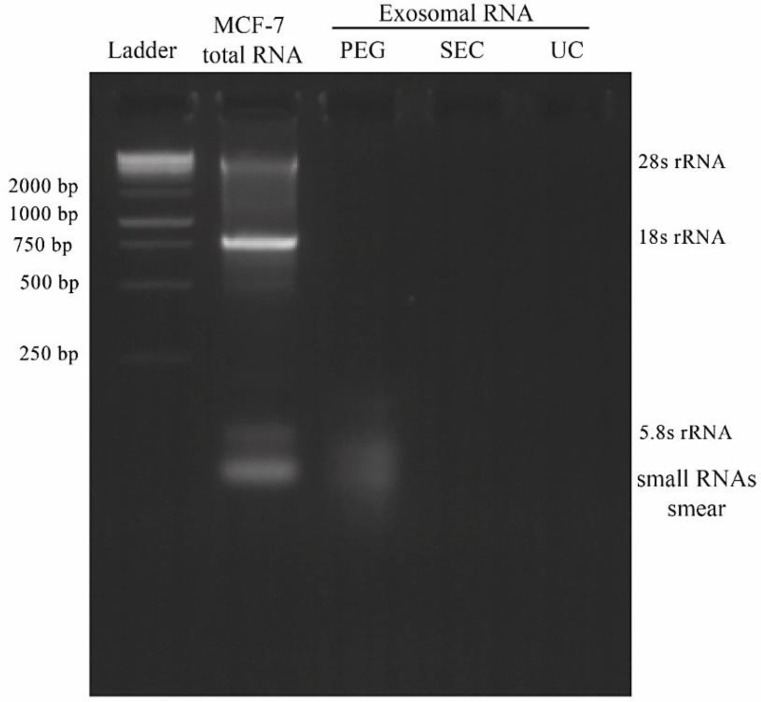
Gel electrophoresis of RNA extracted from MCF-7-derived exosomes isolated by UC, PEG precipitation, and SEC methods on a 2.5% agarose gel. Total cellular RNA was also loaded into gel as a control sample

Nanodrop technique was used to quantify the amount of the extracted exoRNAs and calculate the ratio of absorption at 260 to 280 nm. Overall, the exosomes isolated through PEG precipitation exhibited a higher quantity and purity compared to the other two methods (Table 1). To further confirm the enhanced purity of the extracted exoRNA isolated by the precipitation method, a 2.5% agarose gel electrophoresis was applied. As shown in Figure 3, only the exoRNAs extracted by the PEG-mediated method appeared as a smear on the agarose gel, indicating the presence of diverse types of small RNAs, which might be associated with Exo- miRNAs. However, no apparent RNA bands were observed in the agarose gel for the exosomes isolated by UC and SEC methods.

## DISCUSSION

In the current study, we examined and compared three different traditional methods to define the most appropriate extraction strategy of exoRNA from culture medium. Our findings revealed that the PEG precipitation, compared to the UC and SEC methods, resulted in higher both purity and yield after isolating exosomes derived from MCF. This result was also reported by two previous studies showing that isolation of exosomes using PEG precipitation had a great priority in terms of exosome recovery^[^^16^^,^^17^^]^. For RNA studies, it seems that this method is suitable and simple for the isolation of exosomes. The PEG-based technique is not only easy to manage but also cost efficient^[^^18^^]^. Besides, this method can be performed without high-tech requirements such as ultra- or high-speed centrifugation^[^^19^^]^. The higher the concentration of PEG, the more exosomes were pulled down from the medium. Using PEG precipitation, the isolated exosomes are aggregated and show a large particle size^[^^18^^]^. Consistently, our results revealed a broad peak in DLS experiments with a greater size of the exosomes isolated through PEG-mediated approach compared to the other two methods. Interestingly, the protein concentration was too high to be detected in the exosomes extracted by the PEG-mediated approach when we employed BCA assay, confirming the trapping of the exosomes in 

**Table 1:** Quantification and qualification of the extracted RNA from different samples

**Sample**	**Quantity** **(ng/μl)**	**Absorbance ratio** **(260/280)**
MCF-7 cells	860	2.0
Exosomes (UC)	33	1.8
Exosomes (PEG precipitation)	33	1.9
Exosomes (SEC)	8.65	1.7

PEG particles. However, exosomes obtained by UC and SEC methods had a lower protein concentration than those of the PEG-mediated technique. In agreement with our data, Shieh et al. stated that the PEG-mediated strategy is not desirable for exosome precipitation from protein-rich samples due to the high affinity of PEG to biomolecules such as proteins^[^^18^^]^. Besides, this method showed a higher RNA yield owing to the greater amount of the exosomes obtained. The miRNAs within exosomes are of crucial importance as they potentially represent candidate biomarkers owing to their inherent stability^[^^20^^]^, and thus, the PEG-mediated strategy could provide researchers with easy access to RNA content of the exosomes. 

One of the challenges in RNA studies is the low concentration of the exoRNAs^[^^21^^]^. Herein, we showed that this issue can be overcome by using the PEG-mediated protocol as a large quantity of RNA obtained relative to the UC and Izon column methods. These findings were further confirmed using a Nanodrop assay along with agarose gel electrophoresis. The recovery of exoRNA by PEG precipitation method was greater than that of the other two methods; however, the RNA banding weakened or even disappeared when UC and Izon kit were used. The exoRNA isolated by EXOCIB kit appeared as a smear in the agarose gel, which demonstrates small exoRNAs^[^^22^^]^. Since it has been reported that at least two different approaches should be applied for EVs characterization^[^^23^^]^, we used TEM and DLS analyses to confirm the presence of exosomes in the extracted cell culture media. The results revealed that the isolated nanovesicles were of varying size, resembling that of exosomes. It is worth to mention that exosomes aggregate at high concentrations and show a population with larger particle size in DLS, which were also detected in TEM images. Moreover, it has been revealed that exosomes isolated using the PEG-mediated technique inhibit the cell growth, indicating that the exosomes likely include some chemicals that may suppress the cell growth with no adverse effect on RNA quantity or quality. This observation suggests that each method has its own pros and cons; therefore, researchers have to select the technique insightfully, depending on their purposed use of exosomes for downstream applications^[^^16^^]^.

## CONCLUSION

The PEG-mediated precipitation method provides a user-friendly and inexpensive approach for harvesting exosomes from cell culture supernatant and would deliver an appropriate platform for exoRNA extraction with high yield and satisfying purity. Our future work is directed towards using the exoRNA extracted by the above-mentioned method to further exo-miRNAs study. Considering the broad application of exo-miRNAs in cancer diagnosis, prognosis, and therapy, we suggest this extraction protocol to be used when adequate quantity of miRNAs with appropriate purity are required for a more comprehensive study.

## DECLARATIONS

### Acknowledgments

 In this manuscript, AI has not been recruited in all processes of this work.

### Ethical approval

This study was acknowledged by the Ethics Committee of Pasteur Institute of Iran (ethical code: IR.PII.REC.1399.045).

### Consent to participate

Not applicable.

### Consent for publication

All authors reviewed the results and approved the final version of the manuscript.

### Authors’ contributions

ATG: performed all experimental assays; AN and AR: carried out instrumental analysis; SI: revised the manuscript; MS: supervised the study plan and wrote the manuscript.

### Data availability

All relevant data can be found within the manuscript. 

### Competing interests

The authors declare that they have no competing interests. 

### Funding

This research received no specific grant from any funding agency in the public, commercial, or not-for-profit sectors.

### Supplementary information

The online version does not contain supplementary material.
